# The arrow‐of‐time in neuroimaging time series identifies causal triggers of brain function

**DOI:** 10.1002/hbm.26331

**Published:** 2023-05-20

**Authors:** Thomas A. W. Bolton, Dimitri Van De Ville, Enrico Amico, Maria G. Preti, Raphaël Liégeois

**Affiliations:** ^1^ Connectomics Laboratory, Department of Radiology Centre Hospitalier Universitaire Vaudois Lausanne Switzerland; ^2^ Department of Clinical Neurosciences Centre Hospitalier Universitaire Vaudois Lausanne Switzerland; ^3^ Neuro‐X Institute École Polytechnique Fédérale de Lausanne Lausanne Switzerland; ^4^ Department of Radiology and Medical Informatics University of Geneva Geneva Switzerland; ^5^ CIBM Center for Biomedical Imaging Vaud Switzerland

**Keywords:** arrow‐of‐time, brain dynamics, brain function, causality

## Abstract

Moving from *association* to *causal* analysis of neuroimaging data is crucial to advance our understanding of brain function. The arrow‐of‐time (AoT), that is, the known asymmetric nature of the passage of time, is the bedrock of causal structures shaping physical phenomena. However, almost all current time series metrics do not exploit this asymmetry, probably due to the difficulty to account for it in modeling frameworks. Here, we introduce an AoT‐sensitive metric that captures the intensity of causal effects in multivariate time series, and apply it to high‐resolution functional neuroimaging data. We find that causal effects underlying brain function are more distinctively localized in space and time than functional activity or connectivity, thereby allowing us to trace neural pathways recruited in different conditions. Overall, we provide a mapping of the causal brain that challenges the association paradigm of brain function.

## Graphical abstract

We introduce a novel metric that quantifies the arrow‐of‐time (AoT) in multivariate time series. On functional magnetic resonance imaging data from the Human Connectome Project, we find that AoT strength reflects cognitive status and that unlike functional activity or connectivity, causal effects underlying brain function are highly localized in space and time. We also show that our metric tracks neural pathways recruited during task engagement.

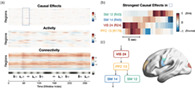

## 1 INTRODUCTION

The advent of functional neuroimaging has provided us with unique insight into the complex spatiotemporal structure of brain function (Damoiseaux et al., 2006). This organization is classically characterized on the basis of association assessments such as functional connectivity (Friston, 2011) that was shown to reflect, for example, cognitive status (Greicius et al., 2003; van den Heuvel et al., 2009) and disease (Anderson et al., 2011; Bassett et al., 2012; Drysdale et al., 2017). However, the limits of this approach in accurately characterizing neural communication and pathways are becoming increasingly appreciated (Reid et al., 2019; Weichwald & Peters, 2021). Therefore, it is crucial to move from association to causal frameworks to improve the interpretation of functional neuroimaging datasets (Siddiqi et al., 2022). For this purpose, various methods have been proposed to extract causal structure from functional imaging time series. They include dynamic causal modeling (Friston, 2009; Friston et al., 2003), multivariate autoregressive modeling (Rogers et al., 2010; Valdés‐Sosa et al., 2005), Granger causality (Barnett & Seth, 2014; Barrett et al., 2010), and more application‐oriented variants of these (Seth et al., 2015).

**FIGURE 1:**
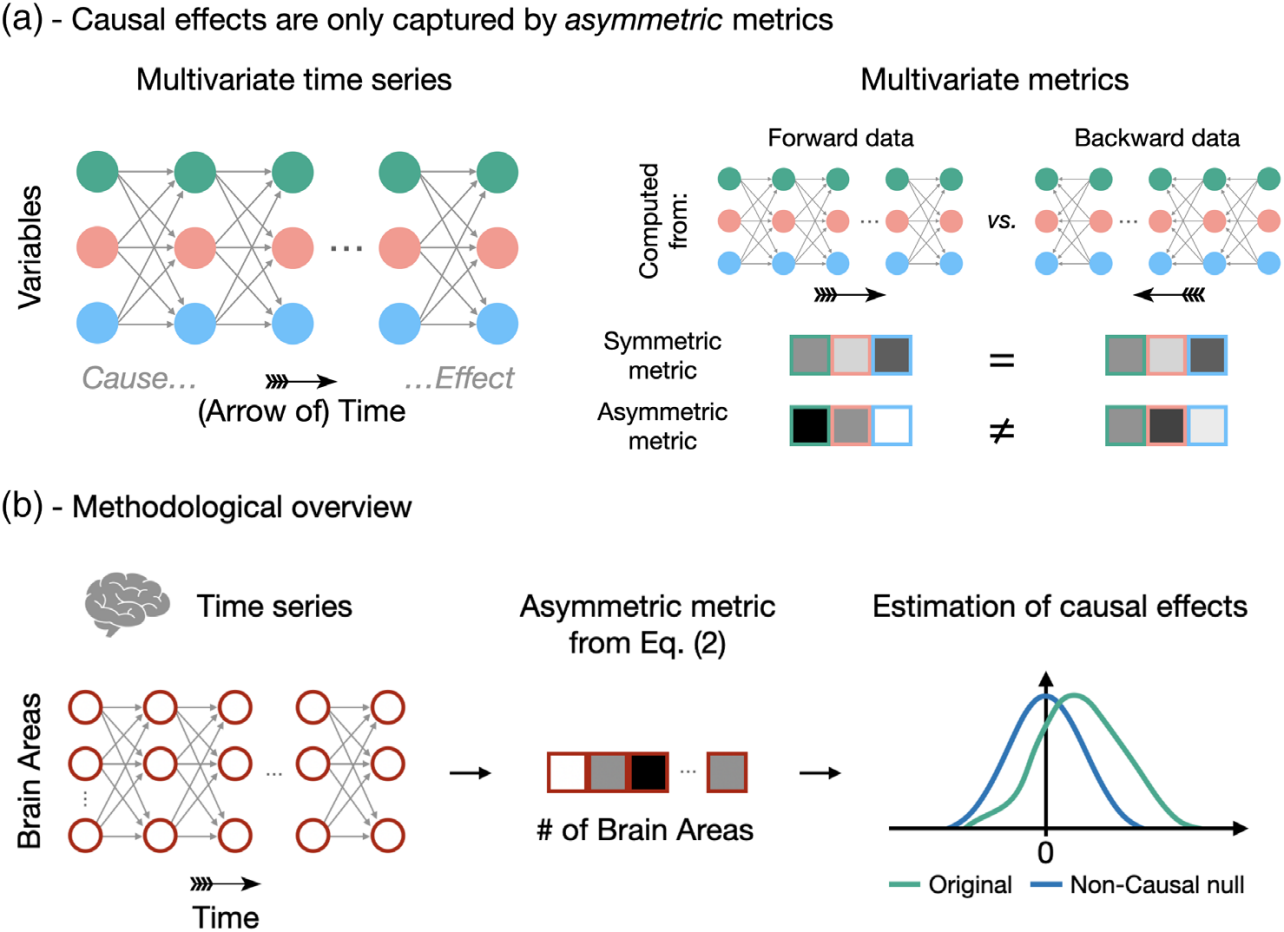
Identifying causal effects in neuroimaging time series using the arrow‐of‐time. (a) Since cause precedes effect, causal effects in multivariate time series cannot be identified from metrics that are blind to the AoT. Such symmetric metrics, for example, mean or average correlation over time points, are equal in forward and backward data. In contrast, asymmetric metrics are different in forward and backward data as they are sensitive to the arrow‐of‐time, thereby bearing the potential of capturing causal effects. (b) We use fMRI time series acquired during resting state and seven different tasks. The AoT signature is evaluated in these time series using Equation (2), and the amplitude of the causal effect is assessed by comparison against null time series with no causal effects.

A shared limitation of these causal discovery approaches, however, is their inability to capture the asymmetry induced by the so‐called *arrow‐of‐time* (Eddington, 1928) (AoT, Figure 1a). Generally speaking, the AoT refers to the fact that, while the physical equations governing the behavior of particles are invariant to time reversal (i.e., they are unchanged if one considers $\tilde{t}=-t$), in practice, time still flows in a preferential direction (Aiello et al., 2008) and we hypothesize that this asymmetry encodes (part of) the causal structure of functional magnetic resonance imaging (fMRI) time series. The AoT has been studied in various fields, including cosmology (Ellis, 2013), quantum mechanics (Castagnino et al., 2005) and thermodynamics (Fröhlich, 2022). In this latter case, in micro‐scale nonequilibrium steady state systems, the establishment of an AoT is tied to the notion of *irreversibility* (i.e., a sequence of events during a process occurs with different probability than the same sequence in time‐reversed order) (Roldán & Parrondo, 2010). Irreversibility is larger when there is more evidence for a forward state sequence as opposed to its backward counterpart (Roldán et al., 2015), and a larger irreversibility goes with a larger breaking of detailed balance, and equivalently, the establishment of an AoT (Lynn et al., 2022a; Lynn et al., 2022b).

Recent studies have pioneered the translation of these micro‐scale concepts to neuroimaging, capitalizing on the fact that irreversibility is then lower‐bounded by the above information‐theoretic criterion (Roldán & Parrondo, 2010). In neurons from the salamander retina, the irreversibility of spiking patterns differed as a function of the visual stimulus (Brownian motion versus naturalistic paradigm) (Lynn et al., 2022a; Lynn et al., 2022b). In the macaque brain, irreversibility in electrocorticography recordings differed between awake, sleep and anesthesia states, when directly contrasting backward and forward state sequence probabilities (Perl et al., 2021) as well as when relying on forward and backward time‐shifted correlation measures (Deco, Sanz Perl, Bocaccio, et al., 2022). In human fMRI data, irreversibility also differed as a function of the performed task, both when quantified probabilistically (Lynn et al., 2021) or when using a machine learning framework to predict the directionality of time series (Deco, Sanz Perl, de la Fuente, et al., 2022).

Here, we introduce a new AoT‐sensitive multivariate metric and apply it to high‐resolution fMRI time series from the Human Connectome Project (Van Essen et al., 2013) (HCP). This metric is a multivariate extension of a previously defined measure (Hernández‐Lobato et al., 2011), and relies on the comparison of residuals of linear models identified from forward versus backward time series. More precisely, we define τ, the AoT strength, as the difference between non‐Gaussianity of the residuals of multivariate autoregressive models of forward time series and backward time series (Figure 1b and Equation (2), details in Section 2). These residuals are expected to be less Gaussian when computed from forward time series (Shimizu et al., 2006), hence we expect τ to be positive. This metric is applied on fMRI data from 100 subjects in the resting state and when performing seven different tasks, thereby providing the AoT strength in each brain region, each condition, and as a function of time during paradigms.

We find that in almost all conditions, the AoT strength averaged over brain regions is positive, that is, the AoT is detected in fMRI time series and shapes their dynamics. Then, we show that patterns of brain regions acting as causal triggers or targets are more sharply localized in space and time as compared to classical activity or connectivity patterns, complementing the “networked‐brain” paradigm that has emerged in recent years (Betzel & Bassett, 2017). Finally, the temporal fluctuations of τ during a task paradigm allowed us to identify a causal pathway of neural activations supporting the task. Overall, our results provide unique insight into the causal structure of brain function by leveraging the asymmetric nature of the passage of time to which almost all classical functional neuroimaging metrics are blind (Pearl, 2000).

## 2 MATERIALS AND METHODS

### 2.1 Data acquisition and preprocessing

We considered S=100 unrelated healthy subjects from the Human Connectome Project S900 data release (46 males, 54 females, mean age = 29.1 ± 3.7 years). We used fMRI recordings acquired at rest and during seven tasks (emotion, gambling, language, motor, relational, social, working memory), for which ethical approval was obtained within the HCP. Our analyses focused on the first of two available resting state sessions, and on each available task session, purely on the left–right phase encoding direction runs. Right–left phase encoding data were examined in supplementary analyses (see Supplementary Material).

To generate regional fMRI time courses, for each run of interest, minimally preprocessed data from the HCP (Glasser et al., 2013; Van Essen et al., 2013) were taken as input. Nuisance signals were first removed from the voxel‐wise fMRI time courses, including linear and quadratic trends, the six motion parameters and their first derivatives, as well as the average white matter and cerebrospinal fluid signals and their first derivatives. In our main analyses, the global signal was also included as a confounding variable. In additional analyses (see Supplementary Material), we contrasted the obtained results to those without global signal regression, and also examined the impacts of performing scrubbing as a final preprocessing step. Voxel‐wise time courses were averaged within each region of a parcellation containing 400 cortical (Schaefer et al., 2018) and 19 subcortical (Fischl et al., 2002; Glasser et al., 2013) areas, for a total of R=419 parcels, and eventually *z*‐scored. To complement these analyses, we also considered cortical atlases containing 200 and 800 regions (Schaefer et al., 2018) (see Supplementary Material).

### 2.2 AoT quantification

To quantify AoT strength across brain regions, we extend a previously defined univariate metric (Hernández‐Lobato et al., 2011) to the multivariate case. First, we fit a first‐order multivariate autoregressive model to concatenated fMRI time series population‐wise (Liégeois et al., 2019), both in the *forward* and in the *backward* directions as shown in Equation (1):

(1) $$\left\{\begin{array}{llll} x_{t} & = & A^{f}\cdot x_{t-1}+\varepsilon_{t}^{f} & \;\text{Forward model}\\ x_{t} & = & A^{b}\cdot x_{t+1}+\varepsilon_{t}^{b} & \;\text{Backward model} \end{array}\right.$$

where $x_{t}$ is of size R×1, $A^{f}$ and $A^{b}$ each have size R×R, and the residuals $\varepsilon_{t}^{f}$ and $\varepsilon_{t}^{b}$ are of size R×1. The model parameters are estimated using ordinary least squares (Stoica & Moses, 2005), and successive samples that originate from separate subjects (owing to the concatenation step) are excluded. Then, the presence of causal effects in different brain regions is assessed by comparing non‐Gaussianity of forward and backward residuals. This was motivated by the fact that residuals of linear models of true cause‐effect links (in this case, the forward model) are more non‐Gaussian than the residuals of the reversed linear models (in this case, the backward model) (Shimizu et al., 2006). Concretely, with T the total number of time points, we define $E^{f}≜{\left\{\varepsilon_{t}^{f}\right\}}_{t=1,\ldots ,T}$ and $E^{b}≜{\left\{\varepsilon_{t}^{b}\right\}}_{t=1,\ldots ,T}$ as the forward and backward error distributions. Regional AoT strength $\tau \left(i\right)$ is then estimated as:

(2) $$\tau \left(i\right)=\underset{\text{Forward non}-\text{Gaussianity}}{\underbrace{{\left[K\left(E^{f}\left(i\right)\right)-K\left(N\left(0,1\right)\right)\right]}^{2}}}-\underset{\text{Backward non}-\text{Gaussianity}}{\underbrace{{\left[K\left(E^{b}\left(i\right)\right)-K\left(N\left(0,1\right)\right)\right]}^{2}}}\;\forall i\in \left\{1,\ldots ,R\right\}$$

where $K\left(\cdot \right)$ denotes the *kurtosis* of a distribution, and $N\left(0,1\right)$ stands for the standard normal distribution. In the case of a marked AoT, non‐Gaussianity of residuals is larger in the forward than in the backward model, and $\tau \left(i\right)$ is positive. From Equation (2) it is seen that in that case, region i is primarily receiving information from the rest of the brain, hence we refer to it as a causal *sink*. By symmetry, we say that if $\tau \left(i\right)$ is negative, brain region i is a causal *source*. Note, however, that a negative value of τ suggests that one model assumption has been violated, for example, due to the presence of an unobserved variable, or due to different delays in hemodynamic responses, and interpretation of negative values of $\tau \left(i\right)$ should be cautious. Finally, we also devised an alternative metric relying on the Kullback–Leibler divergence to quantify AoT strength (see Supplementary Material for details).

### 2.3 Regional AoT patterns

Using $n_{s}^{*}$ samples, regional AoT patterns were extracted for each paradigm of interest. For the compatible tasks, the same process was also conducted after the removal of baseline epochs. To do so, individual binarized paradigm time courses (0 = rest, 1 = task) were convolved with the canonical hemodynamic response function from SPM12, and resulting time points with a value larger/lower than 0.5 were treated as task/rest samples. Of note, since less samples are then available per subject, the obtained AoT estimates gather data from a more extended set of subjects compared to the full recording case.

To study the contribution of separate networks to the AoT patterns, each cortical brain region was assigned to one of seven canonical whole‐brain resting state networks (Yeo et al., 2011) through a majority voting procedure. Positive‐ and negative‐valued AoT contributions were separately quantified.

### 2.4 Significance assessment

To assess AoT significance, comparison was performed to null data for which causal effects were destroyed. For this purpose, for each paradigm at hand, amplitude‐adjusted phase randomization (Theiler et al., 1992) was applied to the original time courses to generate $n_{n}=100$ null realizations. We considered this surrogate procedure in order to destroy causal effects while preserving the original auto‐correlation structure and sampling distribution, including potential non‐Gaussian effects. For each set of null data, using $n_{s}^{*}$ samples, AoT strength was calculated across 100 folds, and the median was taken as an estimate of null regional AoT strength. The mean and standard deviation were quantified for each regional null distribution, and τ was deemed significant if it exceeded the Bonferroni‐corrected $\frac{2.5}{R}\mathrm{th}$ or $\left(100-\frac{2.5}{R}\right)\mathrm{th}$ null percentiles ($\tau^{-}$ and $\tau^{+}$ in Figure 3, respectively).

### 2.5 Software availability

All the scripts used in this work were implemented and tested in MATLAB, versions 2014b, 2020b and 2021a (MathWorks, Natick, MA, USA). They can be freely downloaded from the following link: https://github.com/TiBiUan/AoT_Benchmarking.git. For figure generation, we used the *cbrewer* and *BrainNet Viewer* (Xia et al., 2013) (version 1.7) utilities.

## 3 RESULTS

### 3.1 The AoT characterizes cognitive status

**FIGURE 2:**
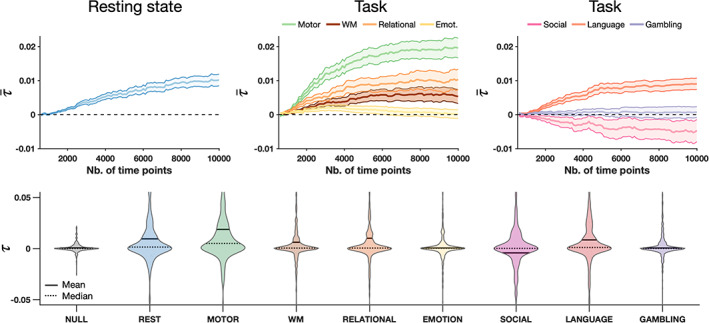
The arrow‐of‐time is detected in functional magnetic resonance imaging time series. *Top*—Estimated AoT strength across regions ($\bar{\tau }$) as a function of the number of available samples at rest (left) and for seven different tasks (center, right), with central lines denoting the mean over regions of interest, and surfaces the standard error of the mean, across subjects. *Bottom*—Distribution of τ across regions using $n_{s}=8000$ time points for estimation in non‐causal surrogate data (NULL, shown here, for indicative purposes, when derived from resting state time courses), at rest, and in seven tasks. Emot., emotion; WM, working memory.

We first evaluate τ in all conditions as a function of the number of time points used. The AoT strength was computed for each brain region across 100 folds in which subjects were randomly ordered and their time courses were concatenated. The median across folds was taken as an estimate of regional AoT strength, and averaging was then performed across regions to derive a whole‐brain AoT heuristic, referred to as $\bar{\tau }$. Figure 2 (top) shows $\bar{\tau }$ as a function of the total amount of considered samples and for all paradigms. In the resting state case (left panel), $\bar{\tau }$ progressively increased as more time points were included, and started to plateau from $n_{s}=8000$ samples, at $\bar{\tau }\approx 0.01$. Thus, when sufficient data is available, the AoT is detected in resting state fMRI time series, confirming the presence of an underlying causal structure.

For task paradigms (middle and right panels), $\bar{\tau }$ also progressively stabilized as more samples were used, but the asymptotic values differed by task: while no sizeable $\bar{\tau }$ was detected for the gambling (purple) and emotion (yellow) tasks, it was negative for the social task (pink), and positive for all others at varying intensities. The largest AoT was obtained for the motor task, at $\bar{\tau }\approx 0.02$. Thus, whole‐brain AoT strength also varies as a function of the cognitive task being performed. The negative AoT found in the social task is surprising and suggests that a model assumption has been violated, for example, the presence of an important non‐observed variable (such as a visual cue), or spatial variation in hemodynamic delays (Buxton et al., 2004).

For subsequent analyses, we focused on the results obtained using $n_{s}^{*}=8000$ samples, as AoT convergence is observed with this amount of data. Figure 2 (bottom) shows estimated AoT strength τ across regions as a violin plot for each paradigm, as well as when quantified from surrogate data having underwent amplitude‐adjusted phase randomization (Theiler et al., 1992), that is, non‐causal null data. In the null case, τ was close to zero for all regions, spanning a narrower range of values than for any paradigm. With the exception of the emotion and gambling tasks, while median τ across regions was close to zero, mean τ was not, denoting that the aforementioned whole‐brain causal effects are induced by a subset of brain areas.

### 3.2 Mapping the causal brain

**FIGURE 3:**
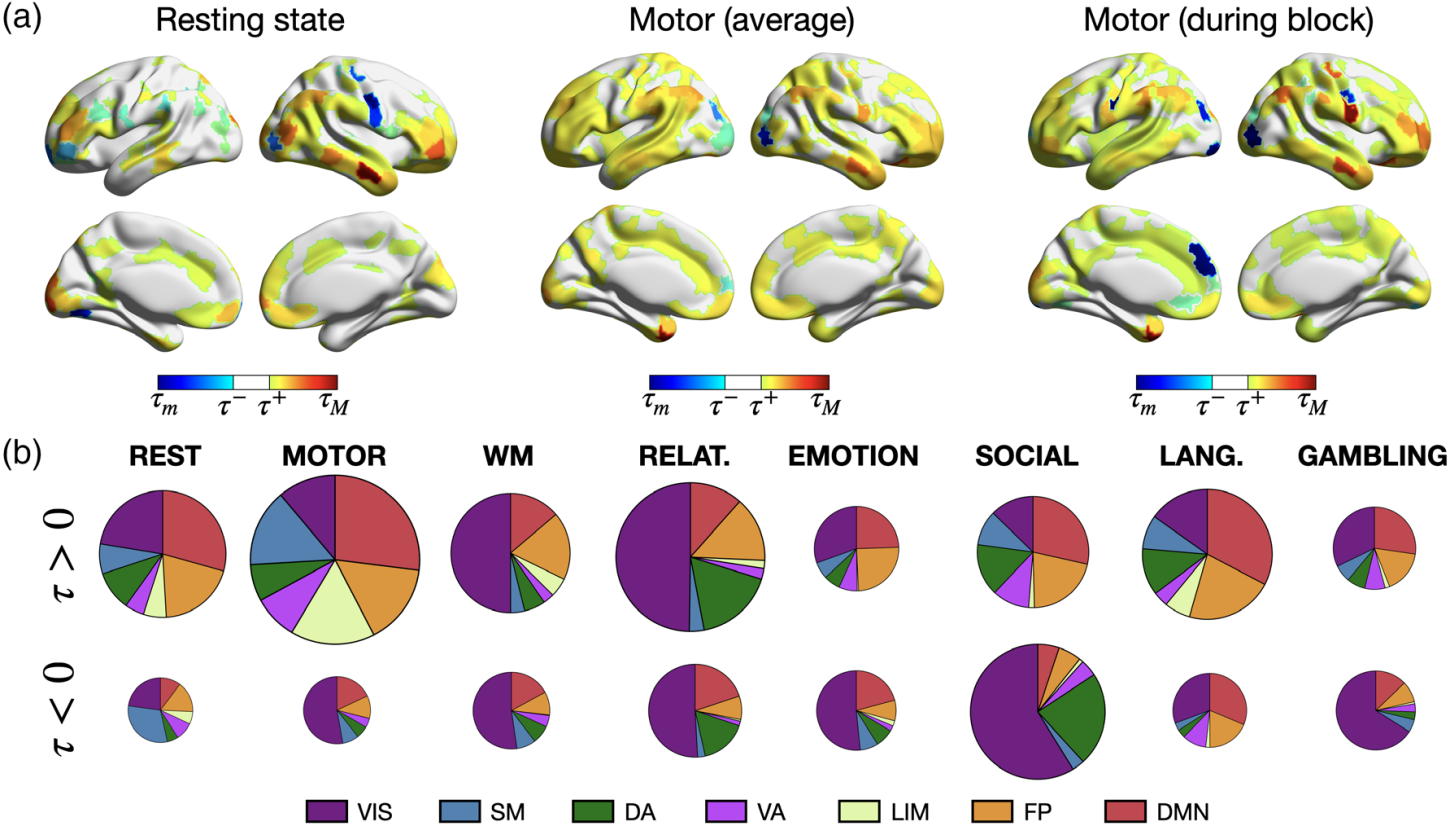
Distinct regional arrow‐of‐time patterns are observed across paradigms. (a) At rest (left), for the full motor task (middle) and when only motor task epochs are considered (right), significant regions in terms of AoT strength. $\tau_{m}$ ($\tau_{M}$): minimum (maximum) value of τ, $\tau^{-}$ ($\tau^{+}$): lower (upper) significance threshold at p=0.05 using Bonferroni correction. (b) For each analyzed paradigm, respective contribution of each of seven canonical networks (Yeo et al., 2011), shown separately for positive‐valued and negative‐valued τ. All areas (including non‐significant ones) are included in this representation. The size of a pie chart is proportional to overall AoT strength in the paradigm at hand.

To determine which brain regions exhibit a significant AoT, we compared them to their respective non‐causal null distributions (Theiler et al., 1992). Figure 3a shows the results at rest (left), and for the motor task when analyzing full recordings (center) or only task epochs (i.e., having excluded baseline periods, right). Figure 3b summarizes network contributions to causal effects in all paradigms where contributions to positive and negative τ were distinguished. From Equation (2), it is observed that a positive τ corresponds to the presence of a causal *sink*, that is, the variable is the target of the causal effect. By symmetry, we associate negative values of τ to the presence of a causal *source*, that is, the variable triggers the causal effect (details on the interpretation of positive and negative AoT values are found in Section 2).

At rest, 184 regions (43.91%) showed a significant AoT, with a mild right lateralization, and positive‐valued τ dominated (130 to 54 negative values). The most significant areas primarily spanned the temporal, prefrontal and parietal cortices, and belonged to the default mode and fronto‐parietal control networks. Some canonical hubs of these high‐level networks showed little significance, such as the posterior cingulate cortex. During the motor task, 284 regions (67.78%) displayed significant causal effects, with no lateralization, and positive values still dominated (214 to 70 negative values). Contributions from the limbic and somatomotor networks were seen in addition to the default mode and fronto‐parietal control ones. When excluding baseline moments, 333 regions (79.47%) became significant, with no evident lateralization, and positive values continued to be more prominent (237 to 96 negative ones). Contributions within the somatomotor cortical stripe became stronger, and some other areas with marked negative values were also newly resolved with regard to the two above cases, such as a low‐level visual region (R218, *VIS18*) and a prefrontal region (R178, *PFC13*). Overall, these results support the presence of stronger causal mechanisms when a subject engages into the motor task as compared to resting state.

More broadly across task paradigms (Figure 3b), negative‐valued τ was primarily observed within the visual network, indicating that it consistently acts as a causal trigger. The only cases where this did not hold true were the language task, which is the only one that involves auditory rather than visual stimulation, and the resting state. Furthermore, the visual network was also dominant in terms of positive contributions for the working memory and the relational tasks, indicating that it also acts as a causal target in these tasks.

### 3.3 From causal maps to neural mechanisms

**FIGURE 4:**
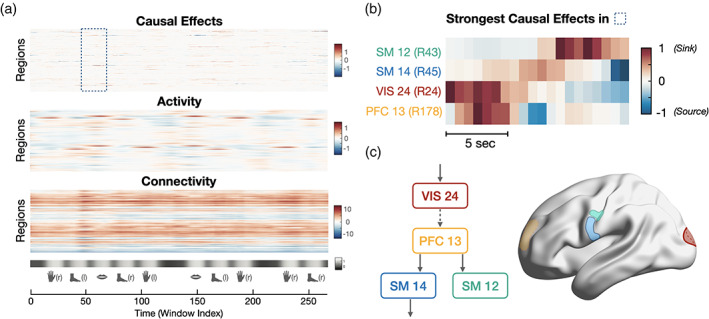
The arrow‐of‐time identifies spatiotemporally localized causal effects in the motor task. (a) Measures of causal effects (τ, top), activity (middle), and connectivity (bottom) during the motor task paradigm. The paradigm consists of movement epochs (left and right hands and feet, tongue), separated by resting blocks. (b) Detailed view of causal effects in left hemispheric brain regions showing the strongest AoT fluctuations in the interval highlighted in panel (a) (tongue movement). Positive values suggest that the region acts as a sink for causal effects, while negative values suggest that the region acts as a source of causal effects. (c) Visualization of the four brain regions in panel (b), together with a putative causal pathway recruited when the subjects start moving their tongue. The dashed line between *VIS24* and *PFC13* means that direct information flow between these two areas cannot be inferred from only the four analyzed regions, and likely involves intermediates.

The differences found between full and task‐only recordings (Figure 3a, middle‐right) hint at strong temporal fluctuations of the AoT. To ascertain this, we performed a sliding window analysis on the motor task paradigm with a window width of W=20 time points slid by one sample until a full AoT strength time course is computed for each region, and using concatenated data from all 100 subjects (Figure 4a, top). Obtained results were contrasted to the activity time courses temporally smoothed with a moving average filter of length W, and to dynamic functional connectivity time courses generated using identical window settings and Pearson's correlation coefficient as functional connectivity measure. In this latter case, we derived a regional measure by summing all functional connections of an area to the rest of the brain within each temporal window.

As expected, clear increases in activity occurred during each of the task epochs in motor regions subserving hand, foot or tongue movement. Connectivity of a given region to the rest of the brain was consistently either positive (denoting a temporally stable regime with more prominent correlation to the rest of the brain), or negative (more prominent anti‐correlation). On the whole, activity and connectivity fluctuations were relatively diffuse in time (spanning full task epochs) and in space (involving many different areas). In contrast, causal effect time courses were highly localized in space (typically only applying to individual regions at any given time point), and occurred within shorter time intervals with fast transition from positive (causal target) to negative (causal source) values.

Figure 4b exemplifies the evolution of causal effects when transiting from baseline to the first tongue movement epoch (see highlighted area in panel A, bottom), for the four left hemispheric brain regions with the largest extent of temporal fluctuations of τ within this interval. Consistent with the paradigm's demands, these regions were motor (*SM12* and *SM14*, for tongue movement), visual (*VIS24*, for parsing the provided instructions), and prefrontal (*PFC13*, to trigger movement execution). When the visual cue is provided to the subjects, *VIS24* becomes a causal sink. Shortly afterwards, *PFC13* becomes a sink, as visual information is treated frontally to make the decision to move. This information is then transmitted to the rest of the brain, as *PFC13* becomes a causal source (see the temporally localized negative values in its time course), while *SM14* and, later on, *SM12* become sinks. Finally, *SM14* further transmits the information and becomes a source to trigger motion. Figure 4c schematically summarizes these observations. Note that regional intensities in temporal fluctuations were also reproducible between the first and the second tongue movement epoch, as evidenced by a significant correlation between both spatial patterns (Spearman's correlation R=0.6,p=0; see Supplementary Material for details).

## 4 DISCUSSION

Here, we introduced a new AoT‐sensitive metric that captures causal effects in multivariate time series. Applied to fMRI data, we showed that causal effects (i) shape brain function in all conditions, (ii) are highly localized in space and time, and (iii) reflect underlying neural mechanisms. These results are found to be robust to head motion, to the use of a different metric of non‐Gaussianity, and to varying processing strategies (see Supplementary Material). While other methods have attempted to assess causality in neuroscience and neuroimaging (Cekic et al., 2018; Friston et al., 2013; Roebroeck et al., 2011; Seth et al., 2015), or to quantify the AoT (Deco, Sanz Perl, Bocaccio, et al., 2022; Deco, Sanz Perl, de la Fuente, et al., 2022; Lynn et al., 2021; Perl et al., 2021), to the best of our knowledge, we are the first to exploit the AoT‐related asymmetry of neuroimaging time series to assess the causal brain.

### 4.1 The AoT provides a new perspective into the causal structure of time series

The term “arrow‐of‐time” has been coined by Sir A. Eddington almost a century ago to *express this one‐way property of time which has no analogue in space* (Eddington, 1928). Rather surprisingly, identifying the AoT from time series is not trivial and most current AoT detection methods rely on deep learning (Deco, Sanz Perl, de la Fuente, et al., 2022; Seif et al., 2020; Wei et al., 2018). Other approaches instead exploit simpler features such as the distribution (Hernández‐Lobato et al., 2011) or the independence (Bauer et al., 2016) of linear model residuals in forward and backward time series. The latter measures, from which we defined τ in Equation (2), also come with a natural interpretation in terms of causality as they leverage causal inference theory to detect the AoT (Bauer et al., 2016; Shimizu et al., 2006). Therefore, the interpretation of τ in terms of causality comes with all causal inference assumptions and guarantees, which is not necessarily the case of other causality detection methods used in neuroimaging studies that encode different forms of causality (Pearl, 2000; White et al., 2011), cf. hereunder.

Identifying causal effects rather than association effects in multivariate time series comes with estimation challenges. For example, it is seen from Figure 2 (see also Supplementary Material for further evidence) that at least ∼1000 fMRI time points are required to identify stable AoT patterns. In contrast, stable patterns of functional connectivity, that is, of correlation, can be identified from as little as around 100 fMRI time points (Van Dijk et al., 2010). Exploiting the non‐Gaussianity of time series through kurtosis also requires cautious estimation of group effects as this metric relates to outliers in a distribution. For this reason, we took several precautions to maximize the stability of our maps: we evaluated our group (original and null) results from the *median* over folds (thus accounting for the selection of different subjects and making our results more generalizable), and adopted the most efficient sample selection scheme after evaluating several candidates (see Supplementary Material). Resorting to non‐Gaussianity of linear models was important in order to unambiguously identify causal structures; indeed, linear‐Gaussian approaches usually only lead to a *class* of possible models equivalent in their conditional correlation structure and from which no unique causal structure can be inferred (Shimizu et al., 2006; Spirtes et al., 2000).

### 4.2 The association brain versus the causal brain

The current perception of brain function has been built from association metrics of functional neuroimaging data, thus probing the ‘association brain’. For example, functional connectivity (Biswal et al., 1995; Friston, 2011; Power et al., 2011), canonical resting state networks (Damoiseaux et al., 2006; Yeo et al., 2011), and most representations of brain dynamics such as (innovation‐driven) co‐activation patterns (Karahanoğlu & Van De Ville, 2015; Liu, 2016), dynamic modes (Casorso et al., 2019), or sliding window‐based states (Allen et al., 2014; Lurie et al., 2020; Preti et al., 2017) are defined from association metrics, for example, correlation, which are blind to causality. By leveraging advances in causal inference, we defined a simple metric that exploits time series asymmetry induced by causal effects. This shift of the methodological paradigm lays the ground to a shift of canonical representations of brain function and dynamics. Furthermore, a causal representation of brain function also comes with promises for the cognitive and clinical use of neuroimaging data as the causal brain is expected to more clearly reflect underlying neural mechanisms (Weichwald & Peters, 2021), as illustrated in Figure 4b,c. Recent neuroimaging endeavors further substantiate this potential: after training a deep learning network to distinguish between temporal segments of forward and backward fMRI time series, Deco et al. (Deco, Sanz Perl, de la Fuente, et al., 2022) not only observed a variable AoT strength (inferred from classification accuracy on unseen data) across cognitive states, but also between healthy subjects and patients suffering from bipolar disorder, attention deficit hyperactivity disorder or schizophrenia. In another study leveraging the same framework on electrocorticography data, de la Fuente et al. (de la Fuente et al., 2023) also revealed that deep sleep and ketamine‐induced anesthesia lowered the differences between forward time series and their inverted counterparts, that is, decreased AoT strength.

Our results show that the topology of the causal brain exhibits strong differences as compared to the association brain. Specifically, the dynamic tracking of the AoT in Figure 4a revealed how remarkably localized it was with regard to functional activation and connectivity. While these two common measures reflect the overall simultaneity in activation across regions, when information has already arrived and been locally amplified (for instance, somatomotor areas in our motor task example), our AoT metric captures the arrival and departure of information. It thus more finely pinpoints the spatial entry and exit points of neural pathways, as well as their exact temporality. As a consequence, time‐averaged representations of the causal brain might be harder to interpret as they destroy the rich temporal structure of causal effects (Figure 3a). In particular, further work will be required to efficiently characterize the causal brain, for example, through causal networks accounting for its specificities.

### 4.3 Differences with respect to popular causal discovery approaches

Here, we wish to elaborate on how our AoT‐sensitive metric differs from existing approaches that seek to extract causal information from neuroimaging data. For an evaluation of their ability, see References (Smith et al., 2011). We specifically focus on Granger causality (Barnett & Seth, 2014; Barrett et al., 2010) and the Linear Non‐Gaussian Acyclic Model (LiNGAM) for causal discovery (Shimizu, 2014; Shimizu et al., 2006; Shimizu et al., 2011) in what follows, as direct links to our methodology exist, but conceptually similar arguments can also be made with respect to other popular methods (e.g., dynamic causal modeling (Friston, 2009; Friston et al., 2003)).

In short, our proposed metric exploits different time series properties, and therefore comes with a fundamentally different interpretation. Similarly to our approach, given multivariate time series $x_{t}$, t=1,…,T, Granger causality relies on a vector autoregressive (AR) representation: $x_{t}=A\cdot x_{t-1}+\varepsilon_{t}$. However, Granger causality is encoded in the matrix of linear coefficients A_,_ 1 whereas our method focuses on the residuals ${\left\{\varepsilon_{t}\right\}}_{t=1,\ldots ,T}$ (cf. Equations (1) and (2)). Therefore, Granger causality can be considered to exclusively exploit linear and Gaussian features of time series, whereas our approach instead harvests their non‐Gaussianity. As for LiNGAM, it exploits the non‐Gaussianity of residuals like our approach, but it does so from a structural equation model in which causal influences are hypothesized to be instantaneous: $x_{t}=B\cdot x_{t}+\varepsilon_{t}$. Furthermore, it comes with the intrinsic limiting assumption that the causal structure of the data obeys a directed acyclic graph (that is, that B is lower triangular).

The other key difference between Granger causality/LiNGAM and our approach is that unlike our metric, these two methods are insensitive to the AoT and the associated concept of irreversibility, as they do not contrast times series to their time‐reversed counterparts. As an insightful illustration for the case of Granger causality, consider the classical forward AR representation of a univariate Gaussian, unit‐norm and centered time series $x_{t}=a\cdot x_{t-1}+\varepsilon_{t}^{f}$, t=1,…,T, where a is a scalar coefficient and ${\left\{\varepsilon_{t}^{f}\right\}}_{t=1,\ldots ,T}$ are normally distributed residuals. When instead identified from backward time series, it can easily be shown that the AR representation still involves the same parameter a; that is, we have $x_{t}=a\cdot x_{t+1}+\varepsilon_{t}^{b}$, where ${\left\{\varepsilon_{t}^{b}\right\}}_{t=1,\ldots ,T}$ are again normally distributed residuals. Two conclusions can be drawn from this example: first, it is not possible to detect the AoT from the regression coefficients of AR models. Second, the interpretation of the regression coefficient “a” in terms of causality requires the prior knowledge of the AoT, as $x_{t+1}$ does not cause $x_{t}$. These fundamental distinctions are summarized in Table 1. In the Supplementary Material, we also provide a network‐level example in which we explicitly show that the effects captured by Granger causality, LiNGAM and our method are different.

**TABLE 1:** Main differences between our method (Equations (1) and (2)), Granger causality and LiNGAM.

Our method	Granger causality	LiNGAM
Autoregressive model	Autoregressive model	Structural equation model
Exploits model residuals	Exploits model coefficients	Exploits model residuals
Non‐Gaussian framework	Gaussian framework	Non‐Gaussian framework
Sensitive to the AoT	Insensitive to the AoT	Insensitive to the AoT

Importantly, it should be emphasized that a precise delineation of the forms of causality detected by different approaches is far from trivial, as illustrated by recent controversies on the topic (Grassmann, 2021). In the present work, we stick to a high‐level overview of salient points, but leave an exact characterization for future work.

All in all, the sensitivity of our metric to the AoT thus bears the promise to capture causal effects to which more classical alternatives, such as Granger causality, are blind. Toward this aim, the exploitation of non‐Gaussian features from time series is a critical asset, since causal effects cannot be determined in a Gaussian setting (Dodge & Rousson, 2001; Fischl et al., 2002). In fact, it is tempting to draw a conceptual parallel with the advent of independent component analysis (ICA) in the extraction of functional brain networks (Damoiseaux et al., 2006): functional time courses were assumed to result from a linear mixture of independent sources, and according to the central limit theorem, this mixture would tend toward a Gaussian distribution. Thus, the maximization of independence (implemented in practice, among other choices, through the maximization of non‐Gaussianity) enabled the extraction of the sources and kick‐started a new chapter of fMRI analysis (Calhoun & Adali, 2006). Similarly, one may hope that relying on non‐Gaussianity in causal frameworks could help unravel the pith and marrow of brain causal effects, compared to Gaussian frameworks that would instead capture a more indistinct mixture of intermingled interactions.

### 4.4 Limitations and further considerations

The proposed characterization of causal effects comes with the assumptions and limitations of the modeling framework in Equations (1) and (2). In particular, we limit our assessment to linear and non‐Gaussian causal effects. This is motivated by the indeterminacy inherent to linear‐Gaussian assessments (Shimizu et al., 2006), but does not mean that causal effects cannot be Gaussian. In future work, it will be important to address to what extent non‐Gaussianity of the residuals is related to nonlinearity of the system.

A good strategy to generalize our framework could be to harvest inspiration from the technical improvements that have been introduced for popular causal discovery approaches: for example, Granger causality has been extended to the nonlinear case (Marinazzo et al., 2011; Runge et al., 2019; Wismüller et al., 2021), while LiNGAM's application was made feasible on chain graphs (Kawahara et al., 2010), in nonlinear settings (Zhang & Hyvärinen, 2009), and in parallel on different datasets sharing the same causal ordering (Shimizu, 2012). Other possible ways forward could be to generalize our autoregressive modeling strategy to a structural vector autoregression model (Hyvärinen et al., 2010), or to consider convergent cross mapping (Sugihara et al., 2012) as an alternative to characterize causal influences.

Robustness to violation of causal sufficiency, that is, the presence of non‐observed variables, would also need to be further assessed (Runge, 2018; Zhang, 2008), potentially by including additional experimental variables of interest such as a record of the visual cue or electrophysiological variables. Then, comparisons across paradigms must be interpreted with caution as while the total number of samples was the same, the length of the paradigms was different. Thus, a distinct number of subjects contributed to the estimates in each case. This directly relates to the question of individual as opposed to population‐wise causal effects, and further work will explore the potential of the causal brain as a subject‐level marker (Finn et al., 2015; Van De Ville et al., 2021). Finally, our framework is directly applicable to other neuroimaging modalities, for example, electro‐ or magneto‐encephalography, but also outside of neuroimaging to any multivariate time series dataset.

## 5 CONCLUSION

Together, our findings suggest that a causal assessment of neuroimaging data indeed provides new insights into the neural mechanisms underlying brain function. More precisely, our mapping of the causal brain hints at key differences as compared to association paradigms of brain function during rest and task, for example, in terms of spatial and temporal localization. In light of this, brain imaging studies have an opportunity to move beyond classical association paradigms and unveil information contained in neuroimaging data to which current metrics are blind.

## Supporting information


**Data S1.** Supporting Information.

## Data Availability

Data were provided by the Human Connectome Project, MGH‐USC Consortium (Principal Investigators: Bruce R. Rosen, Arthur W. Toga and Van Wedeen; U01MH093765) funded by the NIH Blueprint Initiative for Neuroscience Research grant; the National Institutes of Health grant P41EB015896; and the Instrumentation Grants S10RR023043, 1S10RR023401, 1S10RR019307.

## Footnotes

^1^
Precisely, $x_{k}$ is said to Granger cause $x_{l}$ if and only if $A_{l,k}$ is non‐zero (Lutkepohl, 2005).
